# Variation and genetic structure of *Melipona quadrifasciata* Lepeletier (Hymenoptera, Apidae) populations based on ISSR pattern

**DOI:** 10.1590/S1415-47572010005000052

**Published:** 2010-06-01

**Authors:** Marcília A. Nascimento, Henrique Batalha-Filho, Ana M. Waldschmidt, Mara G. Tavares, Lucio A. O. Campos, Tânia M. F. Salomão

**Affiliations:** 1 Departamento de Biologia Geral, Universidade Federal de Viçosa, Viçosa, MGBrazil; 2 Departamento de Ciências Biológicas, Universidade Estadual do Sudoeste da Bahia, Campus de Jequie, Jequie, BABrazil

**Keywords:** Hymenoptera, *Melipona*, genetic differentiation, ISSR markers, population structure

## Abstract

For a study of diversity and genetic structuring in *Melipona quadrifasciata,* 61 colonies were collected in eight locations in the state of Minas Gerais, Brazil. By means of PCR analysis, 119 ISSR bands were obtained, 80 (68%) being polymorphic. H_e_ and H _B_ were 0.20 and 0.16, respectively. Two large groups were obtained by the UPGMA method, one formed by individuals from Januária, Urucuia, Rio Vermelho and Caeté and the other by individuals from São João Del Rei, Barbacena, Ressaquinha and Cristiano Otoni. The Φst and θ^B^ values were 0.65 and 0.58, respectively, thereby indicating high population structuring. UPGMA grouping did not reveal genetic structuring of *M. quadrifasciata* in function of the tergite stripe pattern. The significant correlation between dissimilarity values and geographic distances (r = 0.3998; p < 0.05) implies possible geographic isolation. The genetic differentiation in population grouping was probably the result of an interruption in gene flow, brought about by geographic barriers between mutually close geographical locations. Our results also demonstrate the potential of ISSR markers in the study of *Melipona quadrifasciata* population structuring, possibly applicable to the studies of other bee species.

*Melipona quadrifasciata* Lepeletier, 1836 is a stingless bee distributed along the eastern Brazilian coast, from Rio Grande do Sul to Paraíba, extending westwards inland towards Misiones, in Argentina, and southeastern Paraguay (Moure and Kerr, 1950). Traditionally, two different subspecies are recognized, *M. quadrifasciata quadrifasciata* and *M. quadrifasciata anthidioides*. Their main difference lies in the yellow tergite stripes from the 3^rd^ to the 6^th^ segment that are continuous in *M. q. quadrifasciata* but discontinuous in *M. q. anthidioides* (Schwarz, 1932). The subspecies *M. q. quadrifasciata* is found in the south in the states of São Paulo, Paraná, Santa Catarina and Rio Grande do Sul (Kerr, 1951; Moure, 1975), whereas *M. q. anthidioides* is found from northern and northeastern São Paulo State, eastward up to Paraiba (Kerr, 1951). Populations with a tergite stripe pattern similar to *M. q. quadrifasciata* have been reported in northern Minas Gerais, Sergipe and northeastern Bahia (Batalha-Filho *et al.*, 2009). However, the bees from northern Minas Gerais and northeastern Bahia and Sergipe, differ genetically from *M. q. quadrifasciata* and are similar to *M. q. anthidioides*. Waldschmidt *et al.* (2000), for example, when studying these two *Melipona* subspecies, detected a RAPD marker in individuals from Januária (northern Minas Gerais) that was present in *M. q. anthidioides,* but absent in *M. q.quadrifasciata*. Similarly, Batalha-Filho *et al.* (2009), based on PCR-RFLP analysis of the COI gene, showed genetic similarity between *M. q.anthidioides* and *M. quadrifasciata* from northern Minas Gerais, northeastern Bahia and Sergipe, both with a tergite stripe pattern similar to that of *M. q. quadrifasciata.*

Through population analysis of *M. quadrifasciata*, the difference between the two subspecies could be emphasized (Waldschmidt *et al.*, 2000; Souza *et al.*, 2008), although there are still no reports of studies assessing *M. quadrifasciata* population structuring in itself. Among the techniques that can be used to ascertain population structure, PCR-ISSR has proved to be outstanding in the analysis of natural populations of many plant, fungus, insect and vertebrate species (Wolfe, 2005). Although the importance of this marker in population analysis in insects has been widely shown, studies in the particular case of bees are rare. Berezovskaya *et al.* (2002) detected inter-specific genetic variation in five species of the genus *Bombus*, thereby demonstrating that the ISSR marker could be useful in helping to elucidate problems linked to its taxonomic classification. Paplauskiené *et al.* (2006) detected specific ISSR markers for the subspecies *Apis mellifera carnica* and *Apis mellifera caucasica* that permitted genetic differentiation between the two.

In this study, ISSR polymorphism was assessed and used for characterizing the population structure of *M. quadrifasciata* from different localities in Minas Gerais, thereby constituting the first appraisal of populations with bees of the Meliponini tribe using the PCR-ISSR approach.

Workers from 61 *Melipona quadrifasciata* colonies from eight locations in the state of Minas Gerais, Brazil (Figure 1) were sampled, with one individual per colony being analyzed. Some individuals had different morphological characteristics as regards the abdominal tergite stripe pattern. The locations, number of colonies per location and tergite stripe pattern of the individuals analyzed, can be seen in Table 1.

Genomic DNA was extracted as recommended by Waldschmidt *et al.* (1997). In a preliminary analysis, 93 ISSR primers (UBC Kit) were tested, whereupon 20 were selected based on band reproducibility and definition. The effects of primer concentration (0.05; 0.10 and 0.15 μM), DNA template (10 and 50 ng) and annealing temperature (48-60 °C), were tested. After optimizing amplification reactions, nine primers denominated UBC 807 (54 °C), UBC 808 (54 °C), UBC 834 (48 °C), UBC 836 (54 °C), UBC 840 (50 °C), UBC 842 (56 °C), UBC 848 (56 °C), UBC 856 (48 °C) and UBC 857 (50 °C) were used in the genetic analyses.

The reaction mixture (25 μL) contained 10 ng of DNA, 2.0 μL of dNTPs at 100 μM, 2.5 μL of a 10X buffer, 0.5 U *Taq* DNA polymerase (Prodimol) and 50 pmoles primers. Amplification conditions included initial denaturation at 94 °C for 3 min, followed by 40 cycles of 1 min at 92 °C, 2 min at primer annealing temperature, 2 min at 72 °C, and a final step of 7 min at 72 °C. The reactions were accompanied by a negative control containing all the components, with the exception of genomic DNA. The PCR products were separated by electrophoresis in 1.5% (p/v) agarose gel, and visualized by staining with ethidium bromide (0.2 μg/mL) and photodocumented using the AlphaDigiDoc system.

Amplification products were codified as binary traits according to band presence (1) and absence (0). The percentage of polymorphic loci and genetic diversity (He) were estimated using the TFPGA program 1.3 version (Miller, 1997). Grouping was analyzed by a genetic dissimilarity matrix between the colonies analyzed, based on the Dice index (Dice, 1945), and by the UPGMA method with the aid of the NTSYS program (Rohlf, 2005). The analysis of molecular variance (AMOVA) for studying population structuring was carried out according to Excoffier *et al.* (1992), using the Arlequim 3.01 program (Excoffier *et al.*, 2006). The significance of structuring was tested with 1000 permutations, where P showed the probability of observing a random value equal to or greater than the value observed. Non-differentiation among the locations analyzed was ascertained by exact testing. Genetic diversity and degree of structuring were also analyzed by means of the HICKORY program (Holsinger and Lewis, 2005), using the *free f model*. In this analysis, the H_B_ value was analogous to H_e,_ and the θ^B^ value to Φst of the AMOVA. The spatial distribution pattern based on genetic distance was assessed through the Mantel test using the GENES program v. 2007.0.0 (Cruz, 2007).

Amplifications with clear and reproducible band patterns were available from only nine of the 93 initially assessed ISSR primers (UBC Kit). PCR reactions involving these nine primers resulted in 119 DNA bands, of which 80 were polymorphic (68%). The number of bands per primer ranged from 8 to 16, with a mean of 13 bands per primer, the reactions with UBC-807 and UBC-834 primers presenting the highest number of bands (16). The value of 67% ISSR polymorphism detected in *Melipona quadrifasciata* was considered high and fairly close to that estimated for the subspecies *Apis. mellifera. carnica* and *Apis. mellifera. Caucásica* (66.7%), also when applying ISSR polymorphism (Paplauskiene *et al.*, 2006).

Genetic diversity (H_e_), assuming Hardy-Weinberg equilibrium was 0.20, and the H_B_ estimate based on Bayesian analysis, that does not assume the Hardy-Weinberg equilibrium, was 0.16. Low diversity values have also been reported in studies on other Hymenoptera and were justified by the haplodiploidy system associated to the effective size of the population, social behavior and environmental variation (Graur, 1985).

The grouping analysis (UPGMA) at the level of 0.75 genetic similarities separated the specimens into two large groups. The first group supported by bootstrap value of 99% comprised the samples from Januária, Urucuia, Rio Vermelho and Caeté, whereas the second supported by a bootstrap value of 55% consisted of those from São João Del Rei, Barbacena, Ressaquinha and Cristiano Otoni (Figure 2). The significant correlation between dissimilarity values and geographic distances (r = 0.3998; p < 0.05) determined by the Mantel test, implied possible isolation by geographic distance and the structuring into two large groups might be reflecting this.

Genetic differentiation within populations was probably the outcome of interrupted gene flow, caused by geographic barriers between otherwise geographically close neighbors. This population isolation, as reflected in local genetic structuring, probably arose from the rough geographical topography of Minas Gerais. This hypothesis is supported in studies undertaken by Batalha-Filho *et al.* (2009), who reported that *M. q. anthidioides* is associated with higher altitudes throughout mountain ranges in Minas Gerais, Espirito Santo and Bahia and absent in the lowlands in northern Espirito Santo, southern Bahia and areas in the upper São Francisco river.

UPGMA grouping revealed no genetic structuring associated with the tergite stripe pattern in these bees. The first group supported by a high bootstrap value (bootstrap = 99%) included *M. quadrifasciata* from Januária and Urucuia with continuous tergite stripe pattern and individuals from Rio Vermelho and Caeté in which this pattern is discontinuous. Retained ancestral polymorphism could possibly cause incongruence between ISSR profiles and tergite stripe patterns. Similar results were reported by Batalha-Filho *et al.* (2009) through PCR-RFLP of the COI gene, and in phylogeographic studies that analyzed sequences of the Cyt b gene of these bees (Batalha-Filho *et al.*, 2010). Likewise, Souza *et al.* (2008) investigated patterns of the Cyt b gene, also with PCR-RFLP, whereby they demonstrated a relationship between both RFLP and tergite stripe patterns.

The analysis of molecular variance (AMOVA) showed that the percentage of variation among locations (58.81%) was higher than that within locations (41.19%). The Φst (0.59) showed a high population structuring. The values of the Φst matrix pair by pair between locations confirmed the high differentiation of *M. quadrifasciata* in the state of Minas Gerais (Table 2). Analysis, by applying the Bayesian method, indicated values in line with AMOVA, where the θ^B^ values confirmed the high genetic structuring suggested by the Φst.

AMOVA (Φst = 0.59) and Bayesian analysis (θ^B^ = 0.58) also showed that the two groups were highly structured. Evidence that *M. quadrifasciata* is found structured in the localities assessed in the present study was also reported by Batalha-Filho *et al.* (2010) based on phylogeographical studies.

In short, it was shown that *M. quadrifasciata* genetic structuring, while not expressed in the tergite stripe pattern, could be related to geographic isolation. Furthermore, the potential of the ISSR marker in studies of population structuring in *M. quadrifasciata* was also demonstrated, this possibly constituting a useful aid in the studies of other bee species.

**Figure 1:**
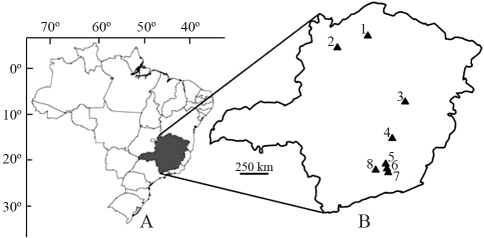
Map of Brazil (A) and the state of Minas Gerais (B) indicating sampled locations of the *Melipona quadrifasciata*. 1- Urucuia (UR), 2- Januária (JA), 3- Rio Vermelho (RV), 4- Caeté (CA), 5- Cristiano Otoni (CO), 6-Barbacena, 7-Ressaquinha (RE), 8- São João Del Rei (SJ).

**Figure 2:**
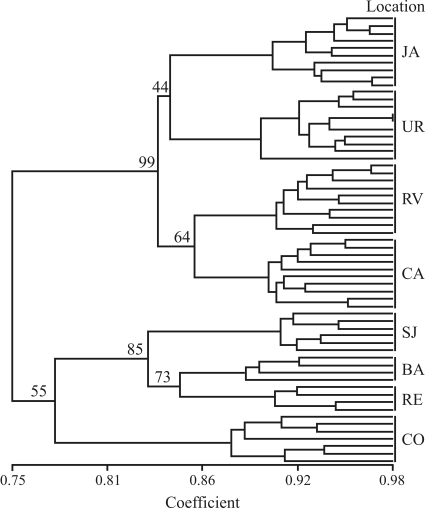
Diagram of genetic similarity obtained by the UPGMA method using the DICE index. Numbers alongside branches refer to bootstrap values (1000 replicates). The column on the right indicates the code of sampled localities.

## Figures and Tables

**Table 1:** Sampled localities, sample size (n) and pattern of abdominal stripes of *Melipona quadrifasciata* from Minas Gerais State, Brazil.

Code /Location	Number of specimens (n)	Tergite stripe pattern
JA-Januária	10	Continuous
UR-Urucuia	10	Continuous
BA-Barbacena	4	discontinuous
RE-Ressaquinha	4	discontinuous
RV-Rio Vermelho	10	discontinuous
CO-Cristiano Otoni	7	discontinuous
SJ-São João Del Rei	6	discontinuous
CA-Caeté	10	discontinuous

**Table 2:** Matrix of Φst values for each pairwise combination among specimes from eight localities based on 80 ISSR loci.

Location	JÁ	UR	CA	RV	SJ	BA	RE	CO
JÁ	0.00000							
UR	0.49610	0.00000						
CA	0.45649	0.50308	0.00000					
RV	0.49604	0.49956	0.38514	0.00000				
SJ	0.68202	0.66954	0.62551	0.57285	0.00000			
BA	0.65050	0.67795	0.66141	0.63524	0.53391	0.00000		
RE	0.68293	0.69245	0.63766	0.60428	0.41472	0.53064	0.00000	
CO	0.67896	0.70995	0.65768	0.63403	0.53504	0.59365	0.37278	0.00000
